# Enhancing the Ammonia Selectivity by Using Nanofiber PVDF Composite Membranes Fabricated with Functionalized Carbon Nanotubes

**DOI:** 10.3390/membranes12111164

**Published:** 2022-11-19

**Authors:** Man Xiao, Yu Shang, Long Ji, Mingwei Yan, Feng Chen, Qingyao He, Shuiping Yan

**Affiliations:** 1 College of Engineering, Huazhong Agricultural University, No.1, Shizishan Street, Hongshan District, Wuhan 430070, China; 2 Technology & Equipment Center for Carbon Neutrality in Agriculture, Huazhong Agricultural University, No.1, Shizishan Street, Hongshan District, Wuhan 430070, China; 3 Key Laboratory of Agricultural Equipment in Mid-Lower Yangtze River, Ministry of Agriculture and Rural Affairs, Wuhan 430070, China; 4 Key Laboratory of Optoelectronic Chemical Materials and Devices-Ministry of Education, Jianghan University, Wuhan 430056, China

**Keywords:** ammonia recovery, CNTs composite membrane, modification, selectivity, electrospinning

## Abstract

Conventional hydrophobic membrane-based membrane distillation (MD) has been applied for ammonia recovery from an anaerobic digestion (AD) effluent. However, the typical hydrophobic membranes do not have selectivity for ammonia and water vapor, which results in high energy consumption from the water evaporation. To enhance the selectivity during the ammonia recovery process, the functionalized carbon nanotubes (CNTs)/polyvinylidene fluoride (PVDF) nanofiber membranes were fabricated by electrospinning, and the effects of different CNTs and their contents on the performance of nanofiber membranes were investigated. The results indicate that CNTs can be successfully incorporated into nanofibers by electrospinning. The contact angles of the composite membrane are all higher than those of commercial membrane, and the highest value 138° can be obtained. Most importantly, under the condition of no pH adjustment, the ammonia nitrogen transfer coefficient reaches the maximum value of 3.41 × 10^−6^ m/s, which is about twice higher than that of commercial membranes. The ammonia separation factor of the carboxylated CNT (C-CNT) composite membrane is higher than that of the hydroxylated CNT(H-CNT) composite membrane. Compared with the application of the novel C-CNT composite membrane, the ammonia separation factor is 47% and 25% higher than that of commercial and neat PVDF membranes. This work gives a novel approach for enhancing ammonia and water selectivity during AD effluent treatment.

## 1. Introduction

With the development of anaerobic digestion technology, the reduction and harmless and resourceful treatment of anaerobic digestion (AD) effluent has become a hot issue. In the raw AD effluent, the total ammonia nitrogen (TAN) concentration varies from 400 to 5000 mg/L based on different fermentation substrates [1,2]. Considering the high volatility of ammonia nitrogen, it is a significant threat to human health and water environment [2,3,4,5]. Hence, it is vital to removal/recover ammonia from the AD effluent. Compared with the typical bio-chemical process to remove ammonia in the form of nitrogen gas, recovering ammonia into fertilizer is an economic and low-carbon-emission pathway [2,6,7,8]. Among various available ammonia-recovery processes, the membrane distillation (MD) based separation process has great potential for ammonia recovery, which is also known as membrane stripping or gas-permeable membrane absorption [1]. The hydrophobic membrane is a solid phase barrier to separate the draw solution from the feed and provide a sufficient gas–liquid area for mass transfer [9]. As only gas vapor is transferred across the membrane pore, MD processes have the advantages of low operating temperature, low operating pressure, and relatively low contamination [1,10,11,12,13]. However, due to the large pore size of the membrane, there is no vapor selectivity in MD processes [14,15]. As a result, the undesired water vapor is transported from the feed to the permeate, which results in high energy consumption and acid requirement [2,16,17]. Therefore, how to increase ammonia selectivity is of importance for reducing energy and chemicals consumption.

During the MD operation, ammonia and water vapor transfer across the membrane under the driving force of vapor partial pressure difference. For typical direct contact membrane distillation (DCMD), elevating the temperature on the permeate side to minimize the temperature difference is beneficial to reduce the water partial vapor pressure difference. This novel isothermal membrane distillation significantly improved the selectivity of ammonia recovery [17]. Similarly, in the sweeping gas membrane distillation (SGMD) process, water vapor transport was also depressed by using high humidity air on the permeate side, in which the feed temperature was 60 °C, and the temperature of the sweep gas was also 60 °C with the relative humidity of 99%. They reported a removal ratio of ammonia reaching up to 85%, while the water-removal ratio only reached 0.1% [18]. Apart from increasing the ammonia selectivity by depression of water vapor transport, increasing the ammonia mass transfer coefficient on the feed side is also important. For instance, most of the studies adjusted the feed pH value to a value higher than 10, hence to the maximum ammonia vapor partial pressure [16,19,20]. Optimizing the operating parameters, such as feed temperature, flow rate and vacuum pressure in the vacuum membrane distillation process is also an option for selective ammonia separation [21,22]. Compared with the application of commercial hydrophobic membranes made from PP, PVDF and PTFE, high-performance membranes have also been fabricated for ammonia recovery recently [1,12,23,24].

Ideally, membranes applied in membrane distillation require high porosity and hydrophobicity, and the pore size ranges from 0.1 µm to 1.0 µm [25,26]. Compared with the traditional methods, such as stretching [27], phase conversion [23], and template immersion [28], membrane preparation from electrospinning exhibits a higher porosity and hydrophobicity [29,30,31], which is satisfied with the requirements of MD [32,33,34,35]. Recently, a high-performance electrospun hollow fiber membrane was fabricated for ammonia recovery [36]. The ammonia mass transfer coefficient was 1.35 × 10^−5^ m/s, and the ammonia selectivity was 7.58 when pH was adjusted to 11. The selectivity was 50% higher than that of using commercial membranes. Notably, the ammonia selectivity can be evaluated by the ammonia separation factor, which is a qualitative index to show the separation degree of ammonia during the process. However, the electrospun hollow fiber membranes not only enhance ammonia transfer, but they also enhance water transfer, and there is a trade-off effect between permeability and ammonia selectivity. To enhance the ammonia mass-transfer coefficient while reducing the water transfer, some fillers with ammonia adsorption properties are fabricated on the membrane surface [12]. The ammonia–nitrogen adsorbents that have been widely studied include [37] bentonite, zeolite, biochar, activated carbon and carbon nanotubes (CNTs). Among them, CNTs have been shown to be an alternative for improving the performance of MD membranes due to good mechanical strength, thermal conductivity, chemical resistance, and hydrophobicity [38]. Moreover, CNTs have a slippage effect, and the smoothness of the nanotube walls, when incorporated on the membrane surface, can increase the vapor flux during MD [24,39]. Carboxylated carbon nanotube immobilized membranes were prepared by the phase conversion method and then used for ammonia recovery; the ammonia flux was 63% higher than that of the commercial PTFE membrane [24]. Importantly, the water flux was kept steady to acquire a high ammonia selectivity. Nevertheless, the membranes prepared by phase conversion generally have low porosity and hydrophobicity [14].

By contrast, electrospinning is an excellent approach for fabricating nanofiber membranes with high porosity and hydrophobicity. What is more, CNTs were also immobilized into nanofiber membranes by electrospinning [14,40], and the membranes were applied for sea water distillation to acquire a flux increase of 33–59% [40]. However, it is still unclear whether the CNTs containing nanofiber membrane have good ammonia selectivity. On the other hand, most of the study used the feed solution with a high pH value to test the membrane performance, which may not conform to the wastewater properties. Importantly, ammonia recovery from wastewater without pH adjustment is satisfied with the requirement of low carbon emissions and a circular economy. Therefore, this work aims to fill the knowledge gap of fabrication and application of adsorptive electrospinning composite membrane for sustainable ammonia recovery by membrane distillation.

In order to fabricate electrospun nanofiber membranes (ENMs) with high ammonia selectivity for AD effluent treatment, the functionalized CNT/polyvinylidene fluoride (PVDF) ENMs were prepared using electrospinning technology, followed by heat-pressed treatment. A comparison study of the hydroxylated carbon nanotubes (H-CNT) and carboxylated carbon nanotubes (C-CNT) on ammonia separation performance was primarily focused on. Additionally, the effect of CNTs concentration and nanofiber diameter on ammonia separation performance was studied. The results provided a sustainable method for ammonia recovery from wastewater using CNT composite ENMs.

## 2. Materials and Methods

### 2.1. Materials

Polyvinylidene fluoride powder (PVDF, Mw = 400,000 g/mol,) was purchased from Macklin (Shanghai, China). The carboxylated carbon nanotubes (C-CNT, inner diameter 5–10 nm, outer diameter 20–30 nm, length 10–30 um, specific surface area 250–300 m^2^/g, purity > 95%) and the hydroxylated carbon nanotubes (H-CNT, inner diameter 5–8 nm, outer diameter 10–15 nm, length 2–8 um, specific surface area 300 m^2^/g, purity > 98%) were purchased from Suzhou Tanfeng Graphene Technology Co., Ltd., Suzhou, China, and Shenzhen Suiheng Technology Co., Ltd., Shenzhen, China. Polyethylene terephthalate (PET) non-wave was served as the support layer for the membrane (Suzhou Shengken Automation Technology Co., Ltd., Suzhou, China). The commercial polyvinylidene fluoride (C-PVDF) based Millipore membrane was purchased from Jintai Technology Co., Ltd. (Beijing, China). Acetone, N, N dimethylformamide (DMF) and ethanol were purchased from Shanghai Hushi Laboratory Equipment Co., Ltd. (Shanghai, China), and were used as received. Lithium Chloride (LiCl, AR grade, >99.5%) was supplied by Sinopharm Chemical Reagent.

### 2.2. Dope Preparation

Table 1 presents the contents of different dope solutions and their codes adopted in this study. For the neat PVDF solution, PVDF powder was dissolved into the DMF/acetone (60/40 by wt%) solvent solution, in which LiCl as an additive with the mass ratio of 0.004 wt%. Then the solution was stirred for 12 h at a temperature of 45 °C, followed by bath sonication for 1 h. Finally, the solution was left at room temperature overnight to remove air bubbles. To prepare the CNT/PVDF solution, the functionalized CNTs were first dispersed into the mixed solvent of DMF/acetone (60/40 by wt%) for 1 h sonication to disperse the CNTs in advance. Then PVDF powder was mixed with the CNTs-DMF/acetone solution by rapidly stirring overnight at room temperature. Before the spinning operation, the solution was sonicated for at least 30 min to disperse the CNTs.

### 2.3. Membrane Fabrication by Electrospinning

**Figure 1:**
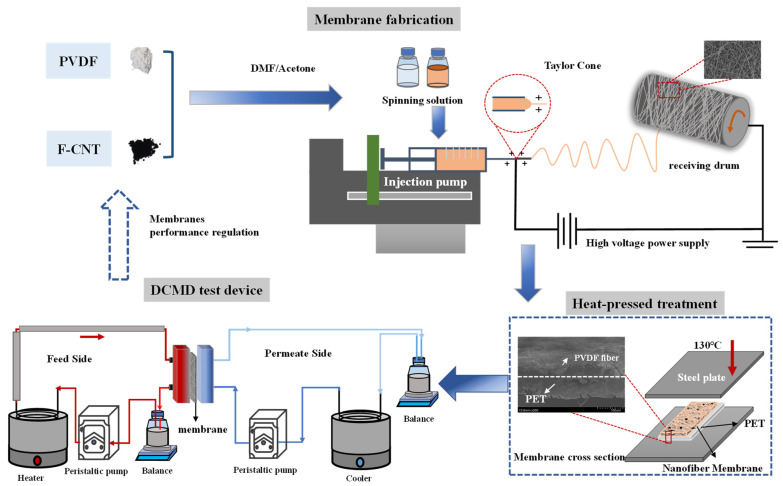
Schematic diagram of the membrane preparation and membrane distillation experiment.

The electrospinning system used in this study is shown in Figure 1. The electrospinning machine was purchased from Foshan Qingzi Precision measurement and control technology Co., Ltd., Foshan, China. Tin foil was first wrapped up in a cylindrical roller to collect nanofibers. Then the dope solution was poured into a 20 mL injection syringe. The voltage, tip-to-collector distance and roller rotation rates were set at 20 kV, 17 cm. 250 r/min, respectively. The feed rate was 0.6 mL/h or 0.8 mL/h, which is set according to the content of CNTs. The temperature and humidity were controlled at 25 °C and 40 RH% during electrospinning. The membrane was peeled off from tin foil, dried at 45 °C temperature for overnight in a hood. Then, the nanofiber composite was hot pressed at 130 °C for 2 h with PET as the substrate.

### 2.4. Characterizations and Measurements

Scanning electron microscope (SEM, JSM-6390LV, NTC, Japan) was used for surface morphology analysis. A surface elemental analysis was performed using a Field emission scanning electron microscope (SU8010,Hitachi, Tokyo, Japan.) with energy-dispersive spectroscopy. The average fiber diameters of the nanofiber membranes were obtained by measuring 100 fiber diameters twice repeatedly using the ImageJ image analyzer. Attenuated total reflectance Fourier transform infrared spectroscopy (ATR-FTIR, IS 50, Thermo Fisher Scientific, Massachusetts, USA) was used to analyze the functional groups on the membrane surface. Membrane hydrophobicity was exhibited by gauges of contact angle (CA) of water droplets with the OCA15EC Video-Based Contact Angle Meter (OCA15E, Dataphysics, Stuttgart,., Germany). Water droplets of about 2 μL were slowly dropped onto the smooth surface of the flat-sheet ENMs through a syringe under ambient temperature, and the contact angles were obtained by measuring three different positions of each sample. The mechanical performance of the samples was measured using a TMS-PRO Texture analyzer (Food Technology Corporation (FTC), USA). The pull up speed was 2 mm/min with the initial pulling force of 0.1 N. The average thickness of the membrane was obtained by measuring the thickness of 8 points of the sample with a digital thickness gauge (CHYQFP12.7, Xingtai Runlian Technology Development Co., Xingtai, China). The porosity of the membrane was measured via a gravimetric method [41]. Specimens with size of 3 cm × 3 cm were cut from the membrane samples and were immersed in ethanol for 2 h. The weights of the dry and wet samples were measured, and the membrane porosity, *ε* was calculated by Equation (1); The liquid entry pressure (LEP) was measured with a homemade test bench using the method published in previous papers [42,43,44]. The average pore size of the membrane was obtained from a Capillary Pore Size Analyzer (CFP-1500AEXL, Washington, DC, USA):

(1) $$\varepsilon =\frac{m_{a}-m_{b}}{\rho_{L}A\cdot l}$$

where *m_a_* and *m_b_* is the weight of wet membrane sample and dry membrane sample. *A* is the effective membrane area (cm^2^), and *l* is the thickness (cm). *ρ_L_* (g/cm^3^) is the density of ethanol.

### 2.5. Ammonia Recovery Performance of Prepared Membrane by DCMD

The DCMD setup used for ammonia separation is shown in Figure 1. The membrane was held tightly between two chambers in the membrane cell (Sterlitech, Washington, DC, USA), with a slot depth of 2.3 mm and slot width 39.2 mm. The total gas/liquid contact area was about 42 cm^2^. In this study, the wastewater targeted is the anaerobic digestion (AD) effluent discharged from biogas plants. AD effluent contains a certain amount of CO_2_ and ammonia nitrogen. Generally, AD effluent is weakly alkaline (pH 7.2 to 8.5) and has good chemical buffering properties. To better compare the ammonia recovery performance of different kinds of fabricated membranes, it is necessary to apply simulated AD effluent to avoid inevitable membrane fouling by AD effluent. The ammonium bicarbonate (NH_4_HCO_3_) solution with TAN concentration of 1000 mg N/L (pH = 8.2) is used as simulated AD effluent in our following experiments. The feed solution was circulated at a flow rate of 150 mL/min derived by a peristaltic pump (Leifu YZ25, Baoding Leifu Fluid Science and Technology Co., Ltd., Baoding, China), and its temperature was set to 65 °C. On the permeate side, 0.05 mol/L H_2_SO_4_ solution was circulated at a flow rate of 75 mL/min under the temperature of 30 °C. Each test was carried out for 240 min, and 5 mL of feed solution was sampled every 30 min. During the experiments, the feed tank was placed on an electrical balance to measure its weight difference (Anheng hengqi Co., Ltd., Dongguan, China). Finally, the ammonia nitrogen concentration in the sample was detected by a flow injection analyzer (AA3, SEAL, Norderstedt, Germany).

### 2.6. Data Analysis

The ammonia–nitrogen separation performance is mainly represented by the total mass transfer coefficient, ammonia separation factor, water flux, ammonia flux and ammonia–nitrogen removal ratio. The mass transfer coefficient of ammonia (*K*_OV_ (m/s)) is an extremely important factor in measuring the ammonia separation performance in the DCMD process, which can be calculated by Equation (2) [12]:

(2) $$K_{\mathrm{OV}}=\frac{V_{0}}{A\Delta t}\ln\left(\frac{C_{0}}{C_{t}}\right)$$

where *V*_0_ is the initial feed volume (L), *A* is the effective membrane area (m^2^), *t* is the time (s), and *C*_0_ (mg/L) and *C_t_* (mg/L) are the ammonia concentrations at start-up and time *t*, respectively.

The ammonia separation factor (*S*_t_) is a qualitative tool to reflect the ammonia separation performance, which can be determined by Equation (3) [2,16]:

(3) $$S_{t}=\frac{{\left(C_{{\mathrm{NH}}_{3}}/C_{H_{2}O}\right)}_{p}}{{\left(C_{{\mathrm{NH}}_{3}}/C_{H_{2}O}\right)}_{f}}$$

where $C_{{\mathrm{NH}}_{3}}$ and $C_{H_{2}O}$ are the ammonia and water molar contents (mol/L) on the permeate side (p) or feed side (f), respectively.

Water flux ($F_{H_{2}O}$, kg/(m^2^ h)) was determined by following Equation (4) [2,16]:

(4) $$F_{H_{2}O}=\frac{\Delta m_{H_{2}O}}{A\cdot \Delta t}$$

where $F_{H_{2}O}$ represents the water flux (kg/(m^2^ h), $\Delta m_{H_{2}O}$ represents the mass of water passing through Δ*t* (h) time.

Ammonia flux ($F_{{\mathrm{NH}}_{3}}$, g/(m^2^ h)) was determined by the following Equation (5) [2,16]:

(5) $$F_{{\mathrm{NH}}_{3}}=\frac{\Delta m_{{\mathrm{NH}}_{3}}}{A\cdot \Delta t}$$

where $F_{{\mathrm{NH}}_{3}}$ represents the ammonia flux (g/(m^2^ h), and $\Delta m_{{\mathrm{NH}}_{3}}$ represents the mass of ammonia passing through Δ*t* (h) time.

The ammonia–nitrogen-removal ratio refers to the ratio of the total amount of ammonia–nitrogen removed from the feed liquid to the total amount of ammonia–nitrogen contained in the feed liquid. According to the ammonia–nitrogen content of the samples taken from the test, the removal ratio of ammonia–nitrogen in each group of tests can be calculated according to the following Equation (6) [2,16]:

(6) $$R=\frac{C_{0}V_{0}-C_{1}V_{1}}{C_{0}V_{0}}\times 100\%$$

where *R* represents the ammonia–nitrogen-removal ratio (%); *V*_0_ represents the volume (L) of the feed solution after running for 10 min in this test; and *V_t_* represents the volume of the feed solution at time *t*. *C*_0_ represents the ammonia concentration (mg/L) of the feed solution after running for 10 min in this test, and *C_t_* is the ammonia concentration (mg/L) of the feed solution at time *t*.

## 3. Results and Discussion

### 3.1. Membrane Surface Chemical Structure 

**Figure 2:**
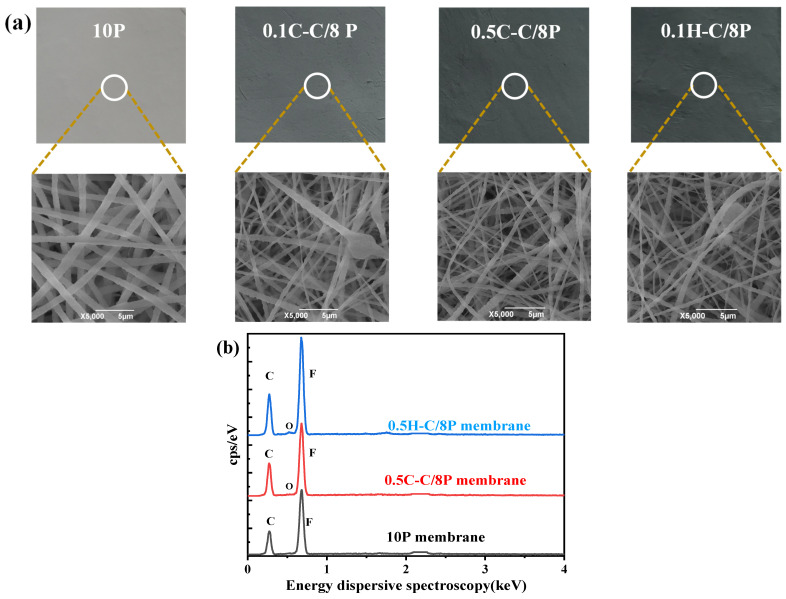

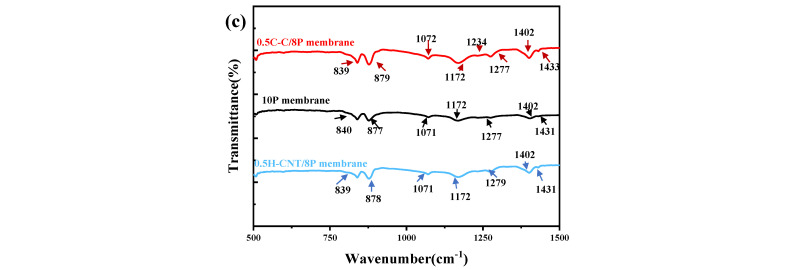
Physical diagram and SEM of different CNT content membranes (**a**), the EDS analysis (**b**), and the ATR-FTIR spectra analysis (**c**) of 10P, 0.5C-C/8P and 0.5H-C/8P membranes.

The image and surface morphologies are shown in Figure 2a. Clearly, the color of the membrane surface changes from light to dark with the increase in CNTs concentrations. The nanofibers are flexible and stacked, which is generally consistent with most of the studies [45]. Compared with the SEM of neat PVDF membrane, it can be found that some nanoparticles are incorporated on the nanofibers, which may result from the CNTs addition [40]. In order to investigate whether the functionalized CNTs were successfully loaded on the ENMs, ATR-FTIR and EDS analyses were performed in this study. Figure 2b shows the results of EDS analysis of 10P, 0.5C-C/8P and 0.5H-C/8P membranes. Compared with neat PVDF membrane, the EDS analysis results show that the composite ENMs contain oxygen elements, which demonstrates the successful fabrication of functionalized CNTs on ENMs [40].

Figure 2c shows the ATR-FTIR spectra of 10P, 0.5C-C/8P and 0.5H-C/8P membranes. All membranes show main absorption peaks at 840 cm^−1^, 877 cm^−1^,1071 cm^−1^ and 1402 cm^−1^, which correspond to CH_2_ rocking or CF_2_ asymmetrical stretching [46], C-C band asymmetrical stretching vibration, C-F band stretching vibrations, C-H bend stretching vibrations [23]. These peaks are attributed to the chemical properties of the PVDF polymer. However, certain peaks are increased after the addition of CNTs, such as 879 cm^−1^ and1072 cm^−1^, which indicate the incorporation of functionalized CNT into the PVDF copolymer matrix [46,47]. CNTs have a slippage effect and a smooth nanotube wall, which can increase the vapor flux during MD [24,39].

### 3.2. Surface Morphology and Hydrophobicity

**Figure 3:**
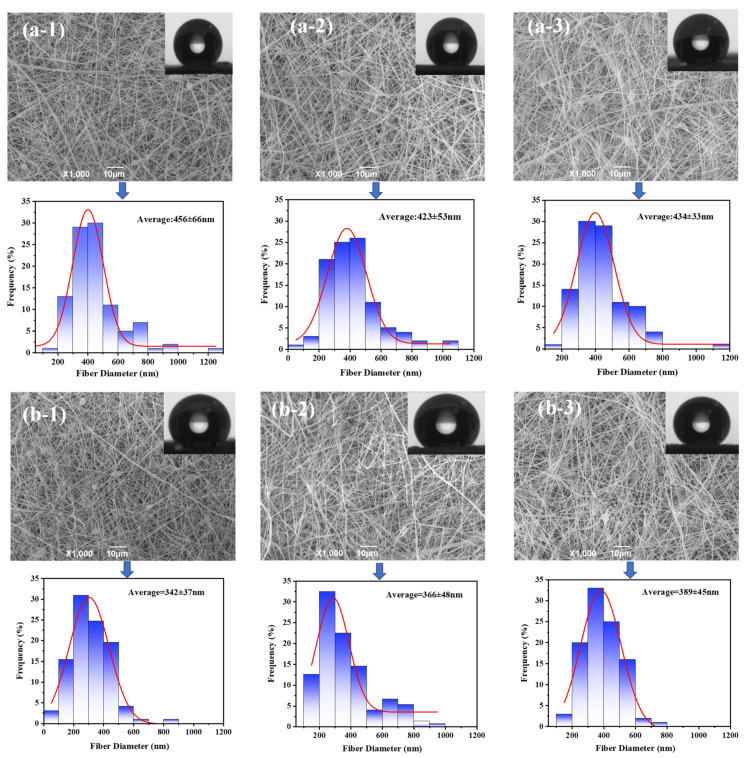
Fiber diameter distribution and water contact angle corresponding to C-CNT (**a-1**,**a-2**,**a-3**) and H-CNT (**b-1**,**b-2**,**b-3**) membranes doped with different CNTs concentrations (0.5 wt%, 0.1 wt%, 0.05 wt%) of 8 wt% PVDF as the substrate, respectively.

The size of the fiber diameter of nanofiber membranes directly affects the membrane performance [48]. In order to investigate the effect of different CNTs contents on the diameter of nanofibers, 100 fiber diameters from SEM images were measured using ImageJ image software (Figure 3). Compared with the modification of C-CNT, the addition of H-CNT can effectively reduce the fiber diameter. Specifically, the average fiber diameter for C-CNT addition group is 437.67 nm, while the value decreases to 365.67 nm for the H-CNT addition group. This is mainly attributed to the CNTs addition which enhanced the electrical conductivity of the spinning solution, thereby increasing the electric field tensile strength to make the fiber finer [49]. To acquire a good mass transfer performance in MD, the H-CNT addition group might better than the C-CNT addition group, because the smaller fiber diameter may bring high porosity and low mass transfer resistance [25,50]. No matter the types of fillers, the water contact angles are all higher than 130°, showing good hydrophobicity for MD. However, the contact angle of the composite ENM decreases slightly with the addition of H-CNT, which is mainly due to the increase in the content of hydrophilic hydroxyl groups [51]. In contrast, the contact angles of C-CNT composite membranes with 8 wt% substrate are all around 138°, and less affected by the C-CNT concentration.

**Figure 4:**
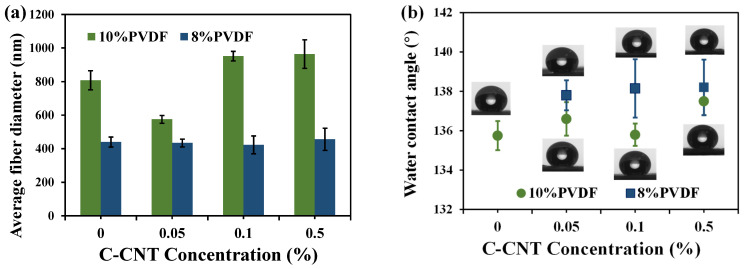
The effect of C-CNT concentration in dope solution on the average fiber diameter (**a**) and water contact angle of the ENMs (**b**).

By adopting a dope solution with 8 wt% PVDF, the effect of CNTs content on the nanofiber diameter is not significant, which is similar to the previous report [46] (Figure 3). According to our initial tests, the optimal pure PVDF concentration for electrospinning in this study was 10 wt%, which could produce bead-free membranes. Therefore, this study also comparatively investigated the effect of incorporating different CNTs contents on nanofiber membranes with different PVDF concentrations. Figure 4a indicates the average fiber diameter of ENMs with different C-CNT content using 10 wt% and 8 wt% PVDFs as the substrate. It is apparent that decreasing the PVDF concentration in the dope solution effectively reduces the fiber diameter of ENMs. Taking the addition of the 0.5C-C/10P membrane as an example, the diameter of the nanofibers decreases from 964 nm to about 456 nm when the PVDF concentration in dope solution decreases from 10 wt% to 8 wt%. The reason may be attributed to the low viscosity of the dope solution [40,52]. Obviously, a lower polymer concentration in the dope solution is favorable for the preparation of fine nanofibers. A lower polymer concentration is also beneficial to fabricate an ENM with a high water-contact angle (Figure 4b). Generally, the smaller the fiber diameter, the larger the contact angle of the corresponding membrane [46]. In the present study, the contact angle of PVDF with low concentration is indeed slightly higher than that of PVDF with a high concentration. However, the contact angles of C-CNTs composite membranes are all higher than 135°, which shows good hydrophobicity and might be beneficial for the MD process. The results suggest that the lower PVDF concentration in the dope solution is favorable for reducing the nanofiber diameter and increasing the water contact angle of the ENMs.

### 3.3. Thickness, Porosity, Pore Size, LEP and Mechanical Property

The characteristics of ENMs and commercial membranes are summarized in Table 2. As for the loose structure of ENMs, the ENMs without heat press treatment have the lowest tensile strength of 0.46 MPa. Such a low mechanical property is not suitable for membrane distillation. To enhance the mechanical properties, ENMs were placed between two iron plates using hydrophilic PET as the substrate for hot pressing. It shows that the tensile strengths of ENMs after heat pressing are all much higher than that of commercial membranes. In particular, the C-CNT-modified membranes have the highest tensile strength (higher than 30 MPa), which is 10 times higher than that of commercial membranes. This is mainly due to the fact that the hot pressing treatment not only establishes cross-linking between the fibers [53], but also promotes the bonding of the fibers to the hydrophilic PET substrate (Figure 1). Obviously, CNTs modified ENMs with a higher tensile strength are suitable for membrane distillation based on the mechanical properties. Nevertheless, the presence of PET would reduce the porosity from 95% to 65.8%, and the thickness would increase from 124 μm to 213 μm. This situation may be harmful to the MD performance. Fortunately, according to Yang’s report [54], the asymmetric membrane may not reduce the flux in MD, but an elevated flux can be acquired by reducing the actual mass transfer route. Herein, the PET layer in ENMs may have an ignorable effect on mass transfer in DCMD.

Table 2 gives the pore size of each membrane prepared with the dope solution containing 0.1 wt% CNTs. The results show that the 0.1C-C/8P membrane has the smallest pore size of 0.59 um, which is an ideal value for MD [26]. It is shown that the pore size of MD membrane is usually between 0.1 µm and 1 µm [34]. Therefore, both H-CNT- and C-CNT-immobilized membranes are suitable for MD. Compared with the commercial membranes, a larger pore size allows for higher permeability, which may also be beneficial for MD. The LEP is related to the high hydrophobicity and maximum pore size of the membrane material [55]. In this study, the difference between the LEP and the average pore size of the membrane before and after hot pressing is not significant. The LEP of the ENM is elevated to 47 kPa after heat pressing, which is similar to the previous studies [14,38,56]. Although the LEP value of ENM is lower than that of commercial membranes, it is still suitable for MD. In summary, the C-CNT composite membranes with 8 wt% PVDF as substrate have high porosity, hydrophobicity, LEP and mechanical strength. This indicates that the ENMs could satisfy the requirements of MD operations.

The basic properties of membranes include membrane fiber diameter, hydrophobicity, mem brane thickness and membrane porosity. To ensure the safe separation of ammonia from wastewaters, membranes need to satisfy some basic characteristics. The above results show that the membranes fabricated in our study can be used in MD for ammonia recovery. In particular, CNTs are fabricated in the membranes successfully. However, no evidence shows that the ammonia selectivity would vary with basic membrane characteristics. Thus, our following results are the primary focus on ammonia-separation performance using different membranes.

### 3.4. Ammonia-Recovery Performance

**Figure 5:**
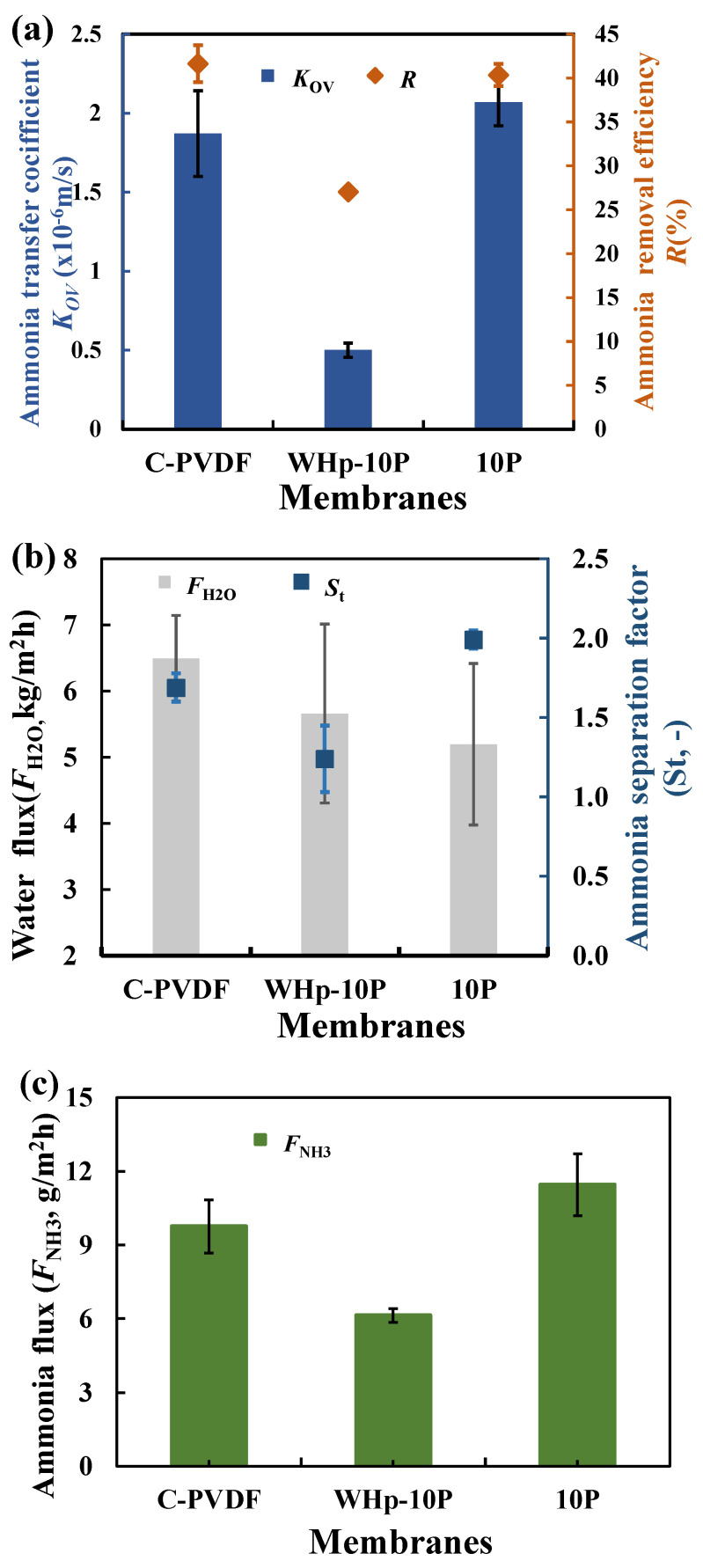
Ammonia-recovery performance of nanofiber membranes without and with hot pressing: total mass transfer coefficient and ammonia and nitrogen separation factors (**a**), ammonia-removal efficiency and water flux (**b**), ammonia flux (**c**).

To investigate the ammonia-separation performance of each composite membrane, DCMD experiments were performed using simulated wastewater without adjusting the pH value. The performance of ENMs was assessed in term of the total mass-transfer coefficient (*K*_OV_) and ammonia and nitrogen separation factors (*S*_t_), ammonia-removal efficiency (*R*), water flux (*F*_H2O_) and ammonia flux (*F*_NH3_). The effects of nanofiber membranes without and with hot pressing on ammonia recovery performance are shown in Figure 5a–c, respectively. The results show that the ammonia nitrogen recovery performance of ENM after hot pressing is significantly improved compared to that before hot pressing and is slightly higher than that of commercial membranes. Specifically, the *K*_OV_ of the membrane after hot pressing is increased by about 3 times compared with the membrane without hot pressing, and 11% higher than that of commercial membranes. The ammonia-removal efficiency increases from 27.04% to 40.35% after hot pressing. This is because hot pressing not only improves the stability of the membrane [57], but also increases the thickness (PET layer addition) and mass transfer resistance of vapor transfer (Figure 5b). However, it is worth noting that the ammonia flux of the membrane is increased from 6.14 g/(m^2^ h) to 11.46 g/(m^2^ h) after hot pressing (Figure 5c). Thus, hot pressing is beneficial for increasing the ammonia-separation performance. Therefore, in order to investigate the ammonia-separation performance of nanofiber composite membranes, the membranes used in the subsequent MD experiments were all hot-pressed at 130 °C.

**Figure 6:**
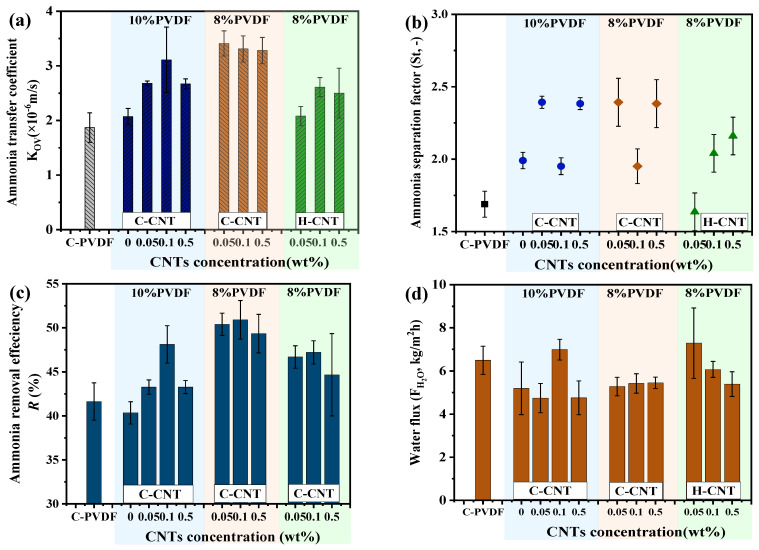
Ammonia-recovery performance using carbon nanotube composite ENMs: total mass transfer coefficient (**a**), ammonia and nitrogen separation factors (**b**), ammonia-removal efficiency (**c**) and water flux (**d**).

The effect of CNT concentration, PVDF concentration and CNT modification types on the ammonia recovery performance are shown in Figure 6a–d, respectively. It is obvious that high water flux is achieved using a commercial PVDF membrane at a feed temperature of 65 °C and a permeate temperature of 30 °C, showing a reasonable MD performance. Based on our previous study, a high water flux was also favorable for ammonia removal. However, the ammonia-removal efficiency is not displayed as well as the water flux by using commercial membranes (Figure 6c,d). This result suggests that membranes with high permeability could provide good water separation performance, but not for ammonia removal. By using the composite ENMs fabricated in this study, good ammonia separation performance with a high ammonia mass transfer coefficient, high ammonia separation factor and ammonia removal ratio can be achieved (Figure 6a,b).

Compared with the neat PVDF membrane, CNTs are key fillers to improve the ammonia-separation performance. Both C-CNT and H-CNT promoted the ammonia separation performance (Figure 6a,b). Specifically, when 10 wt% PVDF was used as the substrate, the largest *K*_OV_ value of 3.11 × 10^−6^ m/s was acquired with the C-CNT concentration of 0.1 wt%. Nevertheless, the membranes fabricated with 10 wt% PVDF have a larger fiber diameter than those using 8 wt% PVDF dope solution. This high PVDF concentration in dope solution leads to a low porosity and a small ammonia mass transfer coefficient (Figure 6a,b). Hence, a fine nanofiber is more suitable for ammonia separation, which is also consistent with previous studies [36].

The highest ammonia separation performance was acquired using C-CNT as the filler, with the ammonia mass transfer coefficient of 3.41 × 10^−6^ m/s, which is about 80% higher than that of commercial membranes and neat PVDF membranes. This is because reducing the PVDF concentration leads to a smaller fiber diameter and membrane pore size. What is more, decreasing the mass concentration of PVDF also corresponds to increasing the C-CNT content to promote ammonia–nitrogen transfer [58]. More importantly, C-CNT is better than that of H-CNT. This mainly because the C-CNT could absorb ammonia, hence promoting ammonia–nitrogen transfer [24,58]. As for the H-CNT composite membrane, which is mainly used to improve the ammonia–nitrogen-removal ratio of the membrane by improving the permeability of the membrane, the results demonstrate that C-CNT composite ENMs with a fine nanofiber are beneficial for recovering ammonia from wastewater without pH adjustment. Apart from adopting the advanced membrane material, optimizing the operating parameters for ammonia recovery is required.

Membrane fouling is a major concern for almost all membrane processes. Due to the high organic content and suspended solids concentration, the membrane is even more easily contained. When treating AD effluent using MD process, the primary contaminants on the membrane are inorganic salts linked with organic fouling. Microbial contamination is also found on the membrane, such as bacteria. Fortunately, although the fouling layer has a high impact on the water vapor transfer, it has less impact on ammonia transfer [59]. However, the effect of contamination on ammonia separation performance still needs to be identified when using carbon nanotube composite ENMs.

## 4. Conclusions

To enhance the ammonia selectivity during the anaerobic digestion effluent treatment process in the membrane distillation process, functionalized CNTs/PVDF nanofiber membranes were fabricated by electrospinning. The membrane characters and their corresponding ammonia separation performance were examined. The results are summarized as follows. (1) The CNTs were successfully incorporated on the nanofibers, and all the ENMs featured with high hydrophobicity and porosity. The highest contact angle of 138° was obtained with 0.5C-C/8P membrane. (2) After hot pressing, the membrane had a higher ammonia recovery performance than that before hot pressing. Specifically, the ammonia transfer coefficient of the ENM increased by about 3 times after hot pressing compared with that before hot pressing, and ammonia separation factor also increased by about 60%. (3) Compared with the H-CNT modified membranes, C-CNT-modified membranes obtained about twice the ammonia separation coefficient and ammonia separation factor. The maximum ammonia transfer coefficient was 3.41 × 10^−6^ m/s, which is about twice higher than that of commercial membranes. Additionally, as the C-CNT content increases, the corresponding ammonia flux of the composite membranes gradually increases. In conclusion, owing to the ammonia-absorption characteristics of the carboxylated carbon nanotube, the C-CNT composite membrane is more favorable for ammonia–nitrogen recovery, showing great potential for anaerobic digestion effluent treatment by MD.

## Figures and Tables

**Table 1:** The compositions used for electrospinning in this study.

Dope/Membrane Code	CNTs (wt%)	PVDF (wt%)	DMF (wt%)	Acetone (wt %)
8P	0	8	55.20	36.80
10P	0	10	54.00	36
0.05C-C/10P	0.05	10	53.97	35.98
0.1C-C/10P	0.1	10	53.94	35.96
0.5C-C/10P	0.5	10	53.70	35.80
0.05C-C/8P	0.05	8	55.17	36.78
0.1C-C/8P	0.1	8	55.14	36.76
0.5C-C/8P	0.5	8	54.90	36.6
0.05H-C/8P	0.05	8	55.17	36.78
0.1H-C/8P	0.1	8	55.14	36.76
0.5H-C/8P	0.5	8	54.9	36.6

Notes: 8P denotes the fabricated membrane with 8% PVDF content; C-C denotes the carboxylated carbon nanotube (C-CNT); H-C denotes the hydroxylated carbon nanotube (H-CNT). Notably, the numbers in front of the letters represent the mass fraction of the component.

**Table 2:** Characteristics of the membranes used in this study.

Membranes Code	Thickness (μm)	Porosity (%)	LEP (kPa)	Mean Pore Size (µm)	Water-Contact Angle (°)	Tensile Strength (MPa)
C-PVDF	204 ± 1.5	74.46 ± 1.10	70	0.22	117.45 ± 1.76	2.33 ± 0.11
WHp-10P	124 ± 18	95.53 ± 2.68	10	1.23 ± 0.66	128.90 ± 1.84	0.46 ± 0.16
10P	213 ± 38	65.80 ± 2.16	10	1.34 ± 0.78	135.75 ± 0.74	16.59 ± 1.43
0.1C-C/10P	183 ± 14	59.37 ± 0.92	10	2.50 ± 1.37	135.80 ± 0.57	30.33 ± 2.11
0.1C-C/8P	214 ± 0.14	63.48 ± 1.81	47	0.59 ± 0.23	138.15 ± 1.48	32.54 ± 3.54
0.1H-C/8P	180 ± 1.9	54.01 ± 1.16	40	0.655 ± 0.22	134.60 ± 0.64	19.12 ± 2.10

Notes: WHp-10P represents the membrane without heat-pressed.

## Data Availability

Not applicable.
